# Effects of Grazing in a Low Deciduous Forest on Rumen Microbiota and Volatile Fatty Acid Production in Lambs

**DOI:** 10.3390/ani15111565

**Published:** 2025-05-27

**Authors:** Raúl Ávila-Cervantes, Pedro González-Pech, Carlos Sandoval-Castro, Felipe Torres-Acosta, José Ramos-Zapata, Mónica Galicia-Jiménez, Ramón Pacheco-Arjona

**Affiliations:** 1 Facultad de Medicina Veterinaria y Zootecnia, Universidad Autónoma de Yucatán, Mérida 97000, Yucatán, Mexico; raul.avila@alumnos.uady.mx (R.Á.-C.); pedro.gonzalez@correo.uady.mx (P.G.-P.); carlos.sandoval@correo.uady.mx (C.S.-C.); tacosta@correo.uady.mx (F.T.-A.); 2 Departamento de Ecología Tropical, Campus de Ciencias Biológicas y Agropecuarias, Universidad Autónoma de Yucatán, Mérida 97000, Yucatán, Mexico; jaramos.zapata@gmail.com; 3 Instituto de Investigación de Genética, Universidad del Mar, Ciudad Universitaria, Carretera Vía Sola de Vega, Puerto Escondido, San Pedro Mixtepec, Juquila 71980, Oaxaca, Mexico; mmgaliciaj@gmail.com; 4 Secretaría de Ciencias, Humanidades, Tecnología e Innovación-Universidad Autónoma de Yucatán, Mérida 97000, Yucatán, Mexico

**Keywords:** rumen, microbiome, bacterial, fermentation, butyrate, 16S rRNA gene

## Abstract

The aim of the present study was to evaluate the effect of grazing the low deciduous forest (LDF) vegetation on the diversity of the rumen microbiome in growing lambs and its relationship with volatile fatty acid (VFA) profiles. After a 35-day indoor acclimatization (stabilization period), the lambs were assigned to two groups: housed (CG, *n* = 4) and grazing (EG, *n* = 4). The grazing lambs had a 14-day habituation period in the LDF (4 h/day) and a further 30 grazing days when fodder intake was observed. Ruminal samples were collected at the end of the stabilization, on day 14 post-stabilization (14DPS), and on day 44 post-stabilization (44DPS). The ruminal butyrate concentration showed a progressive decrease of approximately 23% over the time (*p* = 0.0130). The qualitative composition (*p* = 0.001) and relative proportions of bacteria (*p* = 0.004) in EG-44DPS exhibited a greater diversity, with 107 total genera and 19 unique, significant abundances in 13 genera with a higher presence of *Bacteroidales_RF16_group*, *Lachnospiraceae_ND3007_group,* and *WCHB1-41*. Moreover, significant functional profiles are associated with key metabolic pathways in bacteria and are interconnected by the need to generate energy and biosynthetic precursors and to manage available nitrogen and carbon. Finally, eight bacterial genera were identified as biomarkers correlated with the increase in VFA in EG-44DPS.

## Simple Summary

This study investigated how grazing in a natural low deciduous forest (LDF) affects the rumen microbiome of growing lambs and how these changes relate to their digestion. Grazing led to important changes in the rumen microbial community, increasing the diversity and abundance of certain bacteria known to digest fibrous plant material. These microbial changes were associated with a ~23% reduction in butyrate levels in grazing lambs. In addition, specific bacterial functions involved in nutrient metabolism were more active in grazing lambs. Eight bacterial genera were identified as potential biomarkers of increased volatile fatty acid (VFA) production. These results suggest that allowing lambs to graze on natural vegetation may improve their digestive efficiency by enhancing beneficial microbial communities in the rumen.

## 1. Introduction

The rumen environment is the habitat of various microorganisms, among which are bacteria, archaea, fungi, protozoa, and others [1]. Bacteria play a key role in the degradation of feed and the production of volatile fatty acids (VFAs), the main source of energy for ruminants [2]. The role of bacteria, due to their density and symbiotic relationship, is essential for their mutual survival and crucial for livestock production [3].

The chemical characteristics of feedstuffs are among the main factors directly influencing rumen bacteria diversity [4]. Variations in the chemical composition and nutritional value of the diet lead to differences in the degradation products formed in the rumen, which in turn can modify the structure of the bacterial community [5,6]. Consequently, certain diets promote the growth of specific bacterial groups depending on the chemical nature of the feed [7], ultimately affecting the profile of fermentation end-products [8].

The low deciduous forest (LDF) is a diverse vegetation system that dominates much of Mexico’s tropical landscape, occupying roughly 8% of the national territory and spanning 15 of the 32 states. It hosts a rich plant diversity, with reports identifying up to 2200 species [9,10], among which Fabaceae is the most prevalent family. Extensive research has led to the identification of about 260 plant species with potential use as forage for ruminants. Recent findings indicate that sheep and goats consume up to 61 plant species from this ecosystem [10,11]. Consequently, the LDF serves as a crucial forage base for small ruminant production in many regions of Mexico, where herds frequently rely almost entirely on this vegetation for sustenance.

Some studies have explored interactions between bacterial populations related to efficiency parameters such as VFAs to determine whether grazing affects bacterial communities [12,13,14]. In a study conducted in Qianba, China, on Nanjiang Yellow breed goats, comparative analyses were performed to characterize the rumen microbiota and VFA profiles under grazing feeding systems [13]. Among the three systems, they reported significant differences in total VFA concentrations and the proportions of acetate and butyrate in rumen fluid. Alpha-diversity of rumen bacterial communities was significantly higher in grazing versus housed goats. Likewise, a higher abundance of cellulolytic bacteria such as *Lachnospiraceae*, *Ruminococcaceae*, and *Butyrivibrio fibrisolvens* was found in the grazing groups. They also reported significant correlations between the abundance of various microbial biomarkers and VFA concentrations, suggesting that certain microbial taxa may serve as indicators of grazing-linked cellulolytic bacteria [15,16].

In Mexico, it has been reported that local goats grazing in a semi-arid vegetation type show differences mainly in *Proteobacteria* and *Firmicutes* [17]. The use of mixed native vegetation for grazing is a common feeding strategy in tropical regions and presents a complex nutritional and phytochemical landscape for ruminants. In Yucatán, Mexico, sheep production is primarily based on grazing the native LDF, which includes a wide variety of plant species rich in protein, fiber, other nutrients, and plant-derived functional components. This diverse botanical composition presents unique challenges and opportunities for rumen microbial adaptation and fermentation. Studying lambs grazing in this environment provides valuable insight into how diet-driven shifts in bacterial communities influence fermentation profiles, with potential implications for animal productivity. Therefore, the aim of this study was to evaluate the effects of grazing the LDF vegetation on the diversity of the rumen microbiome in growing lambs and its relationship to VFA production, daily weight gain, and voluntary intake.

## 2. Materials and Methods

### 2.1. Experimental Design

An area (535,000 m^2^) of LDF, a heterogeneous vegetation system of Merida, Yucatan, Mexico, with a hot subhumid tropical climate at 8 m asl, was used. Eight male lambs of the commercial mixed hair-sheep breeds, approximately 3.5 months old, with an average initial weight of 13.95 ± 2.79 kg, were used. The lambs of the experiment were randomly assigned to two groups: housed (CG, *n* = 4) and grazing (EG, *n* = 4), which lasted 44 days. The lambs had a stabilization period of 35 days and were fed to obtain a daily weight gain of ~100 g, the amount being adjusted according to their corresponding live weight during the stabilization period. The ratio of grass to concentrate was 55:45. Subsequently, during a post-stabilization period of 44 days, the CG lambs maintained the same proportion of grass to concentrate ratio, but the amount offered was adjusted weekly according to the lambs’ body weight (Table 1). Grazing in the EG was limited to 4 h per day (07:00–11:00), after which the grazing lambs were housed and fed with grass and concentrate. The ratio of grass, concentrate, and LDF foliage was 25:45:30. The LDF consumption was identified as a likely intake in previous studies conducted on sheep during the rainy season [11]. Ruminal fluid samples were collected at final stabilization, 14 days post-stabilization (14DPS), and at the end of the study 44 days post-stabilization (44DPS).

### 2.2. Estimating Consumption During Grazing

To avoid influencing the grazing behavior of the EG lambs, a 14-day habituation protocol was implemented [18], referred to as 14DPS. To verify the consumption, the method of bite counting [19], adapted to the heterogeneous vegetation of the LDF [20], was used. One EG lamb was observed each day during the four hours of grazing, with a different lamb observed each week.

### 2.3. Collection of Rumen Fluid

Rumen fluid was collected (~30 ± 10 mL) using an oro-ruminal probe adapted to the dimensions of sheep [21] to analyze VFA concentrations and to isolate DNA for metagenomic studies. This collection procedure was performed four hours after the feeding of each group.

### 2.4. Measurement of VFAs in the Ruminal Fluid

The ruminal fluid was filtered through gauze paper to a final volume of 4 mL, and 1 mL 25% metaphosphoric acid was added. The samples were stored at −4 °C until further analysis [22] by gas chromatography [23,24].

### 2.5. DNA Isolation and 16S rRNA Gene Sequencing

From the previous sampling of rumen fluid, 1 ml per lamb was taken, and 500 µL of DNA/RNA Shield (Zymo Research, Irvine, CA, USA) was added for its preservation during its transport to the laboratory. The DNA was extracted using the ZymoBIOMICS DNA Miniprep^®^ Kit (Zymo Research, Irvine, CA, USA) according to the manufacturer’s instructions. Concentration and purity indicators were measured using NanoDrop 2000 (Thermo Fisher Scientific, Wilmington, DE, USA). A ≥ 200 ng DNA per sample was used as input material for library construction and sequencing, using the 16S rRNA molecular marker (V4–V5) on the Illumina NovaSeq 6000 platform (PE, 2 × 150 bp) with ~200 thousand reads per sample, a service provided by Novogene (https://www.novogene.com/us-en/ (accessed on 25 May 2024)).

### 2.6. Statistical Analysis

The assumptions of homoscedasticity of variance and normality of the residuals were tested using graphical methods and the Shapiro–Wilk and Kolmogorov tests. To determine the differences between VFA, DWG, and VI across periods and feed types, a factorial repeated measures analysis was used (*p* ≤ 0.05), and the sphericity criterion was checked by Mauchly’s test (*p* ≤ 0.05) [25,26]. In the sphericity assumption was not met, the Greenhouse–Geisser correction was applied to adjust the degrees of freedom [25,26]. For period comparisons, Bonferroni’s post hoc test was used. Orthogonal contrasts were used to determine the type of parameter behavior (*p* ≤ 0.05) [26,27]. Analyses were performed in Statgraphics 19 software.

### 2.7. Data Analysis

The results of VFA (%molar) in rumen fluid were incorporated into a dataset and matched with the 16S rRNA molecular marker sequence data for each animal and each study group. The quality control of sequences and matching of paired reads was performed using the DADA2 program version 2024.2.0 [28]. Taxonomic assignment was performed with the Naive Bayes classifier and with the q2-feature-classifier add-on using the average classifier [29,30], previously trained with the SILVA 138.1 database (https://github.com/BenKaehler/readytowear (accessed on 10 June 2024)). Sequences were then filtered to exclude annotations with mitochondrial, chloroplast, and eukaryotic features using the filter-seqs plugin. Using a rarefaction curve, a sampling depth value was determined to obtain a uniform number of sequences among the samples, which was used in the diversity analyses.

To analyze the species complexity between GC and EG samples before and after grazing (stabilization and DPS44), beta diversity was calculated using the Jaccard similarity index [31], Sokal–Sneath index [32], Yule index [33], Weighted unnormalized UniFrac [34], and Bray–Curtis dissimilarity [35], considering groups different when *p* ≤ 0.05, using a permuted multivariate analysis of variance (PERMANOVA). The above-mentioned programs were utilized through the QIIME2 v.2024.2 suite [36]. Identification of specific bacterial taxa associated with the two feeding types, data filtering, and graphing were performed using the R package phyloseq version 1.42.0 [37].

Differentially abundant taxa between EG (stabilization) and EG (DPS44) were detected by Analysis of Microbiome Composition (ANCOM) using the ANCOM-BC v2 package in R [38], statistical significance was considered when the false discovery rate-adjusted *p*-value (*q*) was ≤0.05.

Functional prediction between EG (stabilization) and EG (DPS44) was performed using the PICRUSt2 program version 2.5.3 [39], assigning pathways based on the MetaCyc database. Functional profile analysis and visualization were performed with the R package ggpicrust2 [40], using the LinDA method for MetaCyc v.28.5-based data [41]. A correction for multiple testing using the FDR method (*p.adjust* < 0.05) was applied to determine significantly altered pathways between groups, and log2 fold change values were calculated concerning the reference group EG (stabilization).

Finally, to evaluate the multivariate association of microbial community characteristics between EG (stabilization) and EG (DPS44) with VFA concentrations, the R package MaAsLin2 [42] was used, reporting correlations with *p* ≤ 0.05.

## 3. Results

### 3.1. Voluntary Intake and Daily Weight Gain

The EG consumed an average of 41.80 ± 9.25% herbaceous, 20.90 ± 5.29% non-crop grasses, 13.2 ± 1.76% bipinnate shrubs, and 7.1 ± 1.22% creepers in g/kg DM during the DPS44 period. The estimated values of the chemical composition of the diets consumed by both groups during the study are presented in Table 2.

The voluntary intake in the EG increased after 14 days of grazing (*p* = 0.0398) and was maintained at similar levels after 44 days of grazing. In addition, the CG and EG groups were compared at three time points, with significant differences recorded at any period, but only 14 days after grazing (Table S1).

### 3.2. Concentrations of VFAs in Lambs Housed and Grazing LDF over Three Periods

Lambs that grazed for 44 days in LDF presented a decrease in the molar proportions (%) of butyrate (*p* < 0.05), and the orthogonal contrast presents a linear downward trend during the three periods (*p* < 0.05). On the other hand, propionate shows a quadratic trend, increasing its proportions after 14 days of grazing and decreasing after 44 days (*p* < 0.05). However, the molar proportions of propionate between periods are marginally different (*p* ≥ 0.0542) (Table 3). Regarding the group effect, lambs that grazed for 14 days showed a significant decrease in butyrate (*p* = 0.01).

### 3.3. Rumen Microbial Diversity in Lambs Grazing LDF

Fifteen samples were sequenced with ~200,000 reads per sample. After quality control of 3,083,770 raw sequences, 166,565 high-quality sequences (average total length of 317 bp) were obtained (Table S2). The metagenomic sequencing data are available in the NCBI Sequence Read Archive (SRA) repository under accession number PRJNA1241704. The alpha-rarefaction curve showed a depth value congruent with the sample with the lowest number of sequences; the uniform sampling depth resulted in 7618 sequences (Figure S1). A total of 1384 ASVs were identified across all samples.

Species diversity (Yule), proportion of unique characters (Jaccard), and species turnover (Sokal–Sneath) indicated that grazing affected qualitative composition between EG (stabilization) and EG (DPS44) (*p* < 0.05). Abundance (Weighted Unifrac) and dissimilarity (Bray–Curtis) reflected that there were also significant changes in the relative proportions of microbial species due to grazing (*p* < 0.05) (Table 4). The results were similar when comparing the CG (DPS44) and EG (DPS44) groups, except for the Weighted Unifrac metric. In addition, the CG and EG study groups were compared pairwise, with no significant differences, as shown in Table 4. LDF grazing affected the composition and structure of the microbial community, and the differences between periods confirmed that LDF grazing had a unique effect on the rumen microbiota of lambs. The PCoA plots (Figure S2) complement the above results.

### 3.4. Rumen Microbial Community Composition and Functional Profiles in Lambs Grazing the LDF Compared to Housed Lambs

**Figure 1:**
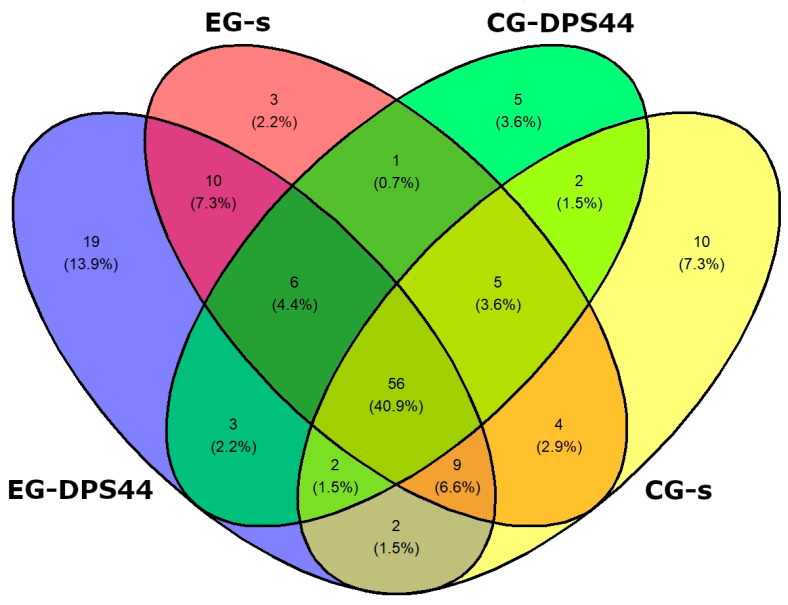
Venn diagram illustrating shared and unique genera among the experimental group in stabilization (EG-s), experimental group 44 days post-stabilization (EG-DPS44), control group in stabilization (CG-s), and control group 44 days post-stabilization (CG-DPS44).

In terms of qualitative composition, 13 phyla, 20 classes, 40 orders, 61 families, and 137 genera were identified in the four groups analyzed. Specifically, 107 genera were identified in EG-DPS44, being the group with the highest richness compared to CG-s with 90, CG-DPS44 with 80, and EG-s with 94. The four groups share 56 genera in common, which are analyzed below with a focus on their differential richness. There are 19 unique genera identified in EG-DPS44, but their proportion in the population is minimal at 0.38% (Figure 1 and Table S3).

**Figure 2:**
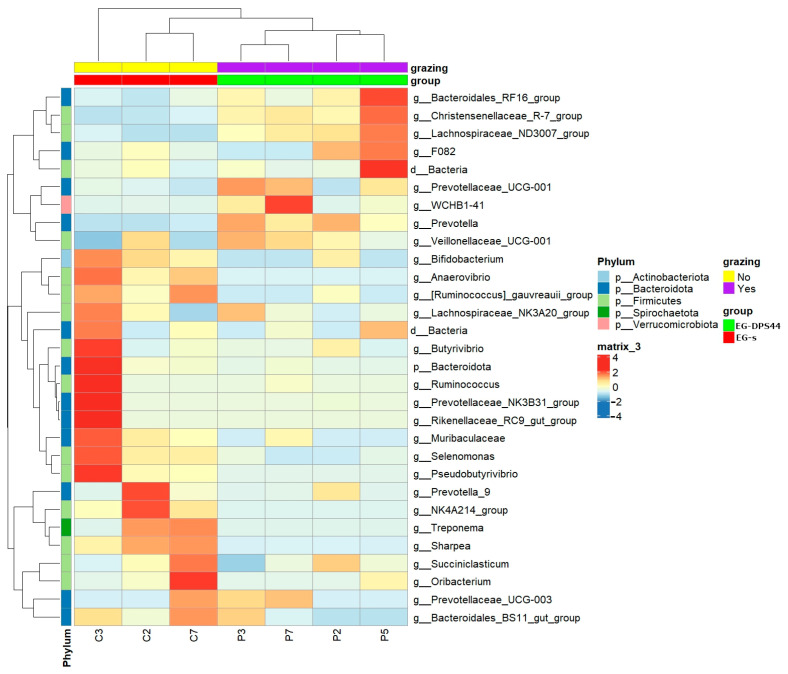
Representative heatmap of the most abundant bacteria at the genus level in the EGs and EG-DPS44 groups. The dendrogram on the left shows the similarity in abundance between phyla, and the one at the top shows the similarity between the elements of each group.

Considering the above results, the different abundance taxonomic levels between EG-s and EG-DPS44 are described. The phylum *Verrucomicrobiota* is more abundant in EG-DPS44, while *Actinobacteriota* decreases. Classes *Kiritimatiellae* and *Alphaproteobacteria* are more abundant, while *Actinobacteria*, *Negativicutes*, and *Bacilli* decrease in grazing lambs. At the order level, *WCHB1-41*, *Christensenellales,* and *Rhodospirillales* increase and *Erysipelotrichales*, *Bifidobacteriales*, and *Veillonellales*-*Selenomonadales* decrease in grazing lambs. At the family level, the *Bacteroidales RF16 group* and *WCHB1-41* are increased in grazing lambs (Table 5). Finally, at the genus level, the strongest differentially abundant candidates correspond to the *Bacteroidales RF16 group*, *Lachnospiraceae ND3007 group*, *WCHB1-41*, *Roseburia*, and *Rikenellaceae RC9 gut group,* with a higher abundance in grazing lambs. While *Sharpea*, *Prevotella_7*, *Bifidobacterium*, *FD2005*, *Erysipelotrichaceae_UCG-002*, *Muribaculaceae*, *Anaerovibrio,* and *[Eubacterium]_ruminantium_group* have lower abundance in EG-DPS44 (Table 5 and Figure 2). Figure S3 shows all elements of the EG-s and EG-DPS44 groups (considered in the ANCOM differential composition analysis) and a heatmap integrating the four groups.

**Figure 3:**
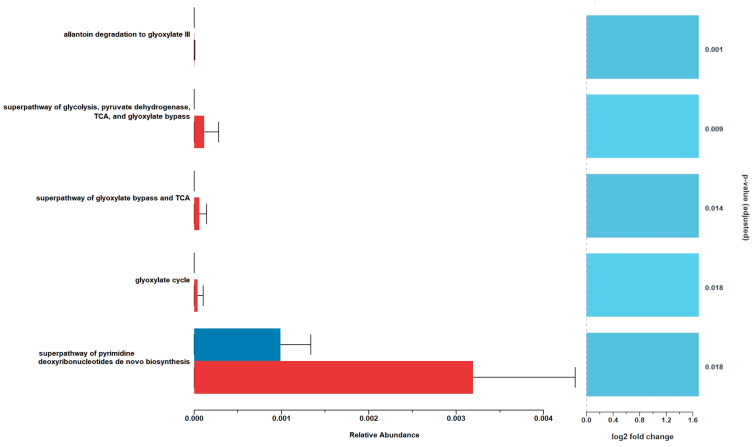
Functional profile of the rumen microbiome data from the EG-s (blue) and EG-DPS44 (red) groups of lambs. The relative abundance of pathways in each group is shown on the horizontal axis. On the far right is the level of statistical significance (FDR) of each pathway. The dotted line indicates a reference point for the change in pathway expression (log2 fold change), with positive values indicating higher expression in EG-DPS44.

The functional profiles with significant differences showed a higher abundance in EG-DPS44 (Figure 3). Allantoin degradation to the glyoxylate III pathway was observed, which degrades allantoin to produce glyoxylate and release nitrogen essential for nucleotide biosynthesis. The superpathway of pyrimidine deoxyribonucleotide de novo biosynthesis requires nitrogen derived in part from the central metabolic precursors, oxaloacetate and D-ribose 5-phosphate. As well as the super pathway of glycolysis, pyruvate dehydrogenase, TCA, and glyoxylate bypass, where glycolysis provides pyruvate, which is converted by the pyruvate dehydrogenase complex to acetyl-CoA, a bridge between glycolysis and the tricarboxylic acid (TCA) cycle, where glyoxylate bypass allows acetyl-CoA to be converted to TCA cycle intermediates without the loss of carbon as CO_2_, which is important in bacteria that use plant compounds such as fatty acids and fiber as carbon sources.

### 3.5. Correlations Between Microbial Biomarkers and VFAs in Lambs Grazing LDF

**Figure 4:**
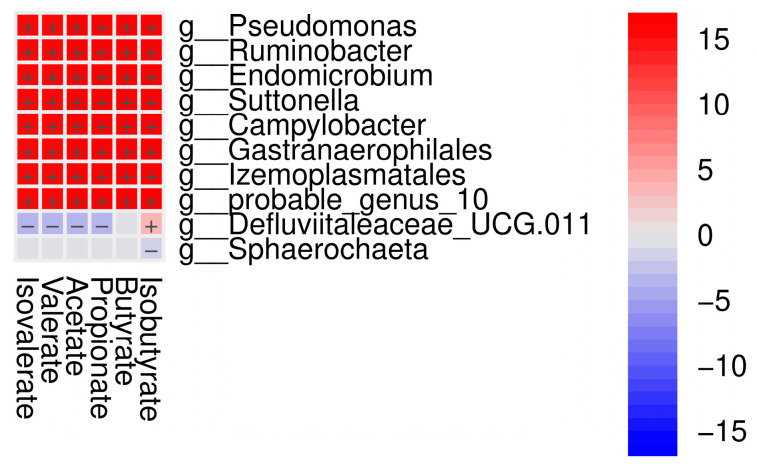
Correlation between bacterial markers at the genus level and %molar VFA, reference EG-s. Correlations with a threshold of statistical significance at *p* < 0.05 were visualized. The red color represents a positive correlation, and the blue color represents a negative correlation.

Correlation analysis between microbial biomarkers and VFAs was performed using 138 variables at the genus level. A positive correlation was found in the genera *Pseudomonas*, *Ruminobacter*, *Endomicrobium*, *Suttonella*, *Campylobacter*, *Gastranaerophilales*, *Izemoplasmatales,* and *probable genus 10* (*p* < 0.001) in the EG-DPS44 group. On the other hand, the genus *Defluviitaleaceae_UCG.011* showed a negative correlation with acetate, propionate, valerate, and isovalerate (*p* < 0.001). In addition, *Sphaerochaeta* showed a negative correlation with isobutyrate in the EG-DPS44 group (*p* < 0.05) (Figure 4).

## 4. Discussion

According to Duncan and Poppi [43], the higher intake of herbaceous plants during grazing observed in the EG-DPS44 group may be related to their feeding strategy [44] and the body size of the lambs. Janis (2008) and Jaimez-Rodríguez et al. (2019) [45,46] point out that the choice of food can be attributed to the mixture of plants available in the area and their ecological structure. The high consumption of shrubs (~26%) could be important for the adaptation and proliferation of ruminal bacteria observed in EG-DPS44, possibly related to better utilization of nutrients, as shrub vegetation in LDF has been classically reported to have high protein, fibrous contents, and low energy content [11,47,48].

Butyrate proportions in the EG lamps showed a significant linear decrease (*p* = 0.0102) across the periods analyzed. Butyrate decreased by approximately 21% from period 1 (16.50 ± 0.89%) to period 2 (13.05 ± 1.57%), and by around 3% further from period 2 to 3 (12.68 ± 0.27%) (Table 3). The butyrate is produced by fiber fermentation [49,50], so its decrease could be related to the high indigestible fiber intake characteristic of LDF plants [51]. The above agrees with Guo [13], who reported the decrease in VFAs, including butyrate, in goats grazing in an area with subtropical climate shrubs mostly composed of grasses such as *Imperata cylincrica*, *Miscanthus sinensis,* and *Deyeuxia arundinacea* and shrubs of *Lespedeza bicolor* and *Indigofera amblyantha*, rich in poorly fermentable fibers. On the other hand, propionate was not significant (*p* = 0.1075), but it exhibited a marginally significant quadratic behavior (*p* = 0.0513), showing a moderate increase during the EG-DPS14 (second period), due to a higher consumption of herbaceous plants that may present a lower availability of structural carbohydrates; in addition, the chemical composition of these plants presents a lower amount of indigestible fiber during the rainy season than in any other time of the year [48].

In terms of bacterial diversity, grazing the LDF affected both the composition and structure of the microbial community in EG-DPS44 lambs. In agreement, it has been found that the diversity of rumen bacterial communities was significantly higher in grazing goats compared to housed goats [13]. In the composition of the EG-DPS44 lambs, a notable increase was observed in the phylum *Verrucomicrobiota*, which increased by approximately 29.6 times (*p* = 3.08 × 10^−05^), as well the class *Kiritimatiellae*, and the order, family, and genus *WCHB1-41*, both of which increased by around 32 times (*p* = 3.08 × 10^−05^, and *p* = 3.10 × 10^−05^, respectively) was notable. In this regard, a study of the gut microbiome of Asian elephants (*Elephas maximus*) revealed that the bacterial taxon *WCHB1-41_c* is progressively enriched in free foraging environments, and tends to decrease with the increasing degree of captivity, similar to what was observed in the grazing (EG-DPS44) and housed (CG-DPS44) groups, respectively. In addition, it was identified that the major functions of *WCHB1-41_c* progressively increase from fully captive to wild populations [52], possibly in part due to a heterogeneous vegetation diet, equivalent to that of the EG-DPS44 group, but composed of other plant species. In another investigation of the relationship between diet and microbiota in yaks (*Bos grunniens*), a significant increase in *Akkermansia* and uncultured *Eubacterium WCHB1-41* was detected at low nitrogen and energy intakes during a cold season, suggesting a response to a diet high in fiber and low in protein [53]. Fiber-rich diets increase the thickness of the intestinal mucus layer, thus improving barrier function [54]; it has been reported that *Eubacterium WCHB1-41* participate in the degradation of mucins, producing short-chain fatty acids (SCFAs) (also known as VFAs), which serve as a nutrient source for other bacteria and host cells [55]. On the other hand, Wei et al. [56] demonstrated that supplementation with *Astragalus membranaceus* root extract in yak increased the proportion of *WCHB1-41_c*, which improved the final weight and average daily gain. The increase in the genus *WCHB1-41* and the increase in DWG in EG-DPS44 are consistent with the above study and could be related to the high consumption of herbaceous plants (41.80 ± 9.25%), such as the plant *Tetramerium nervosum* (34.15 ± 14.62%), which has the highest consumption. A notable decrease in *Actinobacteria* was observed in grazing lambs, with its abundance reduced by approximately 22 times (*p* = 8.69 × 10^−06^), which is related to fiber degradation [57]. Decreases were also seen in *Negativicutes,* important in diets with low energy intake [58], in the *Muribaculaceae* genera, related to the formation of the rumen and intestinal mucosa [59], and in *Anaerovibrio*, important in the degradation of lipids and fibers [60], and these may be due to their replacement by the growth of *WCHB1-41* bacteria, which is better adapted to the consumed substrate in the LDF.

The genus *Bacteroidales RF16 group*, associated with protein and amino acid degradation [61], endotoxin reduction and inflammation prevention [62,63], and homeostatic functions in the rumen and intestine and energy supply [58,61] as well as the genus *Lachnospiraceae ND3007 group,* associated with VFA formation [64] and a possible relationship with lysine and methionine synthesis in the rumen [65,66], are both reported in the study by Li [67]. They carried out a study using calves fed diets with different levels of maize silage, where the abundance of the *Bacteroidales_RF16group* significantly increased and was positively correlated with propionate production. A similar trend was observed in grazing lambs, where the abundance increased by approximately 23.8 times (*p* = 8.79 × 10^−22^). On the other hand, the genus classified in the same study as *Unclassified_Lachnospiraceae* showed a decrease and a positive correlation with acetate production. This contrasts with our findings, where the abundance of this genus increased in grazing lambs by approximately 9.3 times (*p* = 2.6179 × 10^−07^). Schären et al. [68] in their work on Holstein dairy cows fed diets composed of maize silage, forage, and concentrate feed, reported an increase in microorganisms of the order *Bifidobacteriales* and related it to the increase in butyrate. In the grazing lambs, the microorganisms belonging to this order (*p* = 8.779 × 10^−06^) and the proportions of butyrate decreased, possibly due to maize silage having a better composition of digestible fibers, contrasting with those consumed by the lambs in EG-DPS44.

Like the *Bacteroidales RF16 group*, the genus *FD2005* has been related to protein degradation and positively associated with isovalerate formation in the rumen fluid of cows [69]. Something similar occurs with the *Lachnospiraceae ND3007* group and the genus *Erysipelotrichaceae_UCG-002*, which also shows a close relationship with the total concentration of VFAs in lambs of the Hu breed [70]. Both genera decrease in abundance in grazing lambs, and possibly also due to a functional substitution or displacement by better adaptation of the *Bacteroidales RF16* group and *Lachnospiraceae ND3007* group to the substrate ingested by the host, influencing the production of key VFAs that affect animal performance and health.

Among the genera associated with VFA concentrations and potential biomarkers in lambs grazing the LDF are the bacteria *Endobacterium* and the genus *Pseudomonas*. *Endobacterium* is not a bacterium characteristic of the rumen but has been reported to be present in plants and to control the proliferation of phytopathogenic fungi such as *Rhizopus microsporus*. Some members of the genus *Pseudomonas*, such as *Pseudomona aeroginosa*, in enriched cultures of goat rumen fluid, inhibit methane production in nitrogen metabolism and feed digestibility [71].

Also, the genus *Ruminobacter* is important in the formation of succinic acid, acetic acid, and lactate [72] intermediate forms and is important for the formation of VFAs [73]. Meanwhile, *Suttonella* is important in the fermentation of simple sugars [74], and its abundance is related to body weight and body fat content in sheep [75]. *Gastranaerophilales* can degrade sugars, especially hemicellulose [76], which may be related to the rapid metabolism of butyrate, promote digestion, and be a source of vitamins B and K [77]. The *Probable_genus_10*, negatively correlated with glycolysis and gluconeogenesis [78], and is involved in primary and secondary food degradation [79]. *Sphaerochaeta* probably has pectolytic activity and the ability to produce acetate [80]. *Defluviitaleaceae_UCG.011* is directly linked to propionate production [81]. Collectively, these genera are linked as active participants in the formation of VFAs, going through substrate degradation, intermediate phases, as well as probably a direct participation in their synthesis.

Prediction of EG-DPS44 functional profiles shows increased expression in the superpathway allantoin to glyoxillate III. This pathway produces urea and glyoxylate in addition to ammonia and CO_2_ [82]. Glyoxillate is critical for the condensation of propionyl-CoA to α-hydroxyglutarate [83], and its pathway is considered an alternative to produce propionate [84]. In addition, the ammonia produced during this pathway may be important for microbial protein synthesis [85]. Ammonia is essential for fiber degradation because it is an essential component for the growth of bacteria with cellulolytic capabilities, which mainly use ammonia as a nitrogen source [86].

In addition, the superpathway glycolysis, pyruvate dehydrogenase, TCA, and glyoxylate bypass increased in EG-DPS44 lambs. Its importance lies in the presence of alternative steps in TCA that release CO_2_, allowing the conservation of carbon sources with two molecules, such as certain fatty acids [87,88]. In a study of metabolic changes in serum and milk of Holstein cows, it is mentioned that glyoxylate and dicarboxylic acid metabolism uses intermediates such as isocitrate and α-ketoglutarate, which participate in the TCA cycle to regulate amino acid metabolism [89].

The glyoxylate cycle pathway participates in the synthesis of macromolecules by using acetyl-CoA as the sole carbon source. The substrates used by this pathway are alcohols, esters, alkanes, and fatty acids [90]. On the other hand, it could potentially be linked to propionate production, although in limited amounts [91].

Finally, the de novo pyrimidine super pathway is a very important pathway in DNA synthesis [92]. This pathway has a high energy demand and could be related to the previously mentioned pathways. A study conducted by Kheirandish et al. [93], with a metabolomic approach in vitro conditions with rumen fluid from cows, demonstrated that sources with easily degradable carbohydrates can reduce the expression of this pathway. The consumption of heterogeneous vegetation is low in energy content, which could be related to a higher expression of this pathway in grazing lambs and the proliferation of specialized bacteria, which may affect the metabolic pathways of strategic importance for energy synthesis and maintaining homeostasis in ruminants.

## 5. Conclusions

This study described the changes in different parameters of lambs when changing them from a confined feeding system to grazing in the LDF. The patterns of ruminal fermentation were modified, possibly attributed to the chemical composition of the heterogeneous vegetation of the LDF. The characterization of the compositional change in the bacterial microbiome indicates that a qualitative and proportional structural change occurred, with greater relevance in the latter. An increase in bacteria capable of fermenting structural carbohydrates was observed, alongside a decline in bacteria associated with the degradation of substrates other than the vegetation of LDF. Furthermore, there is greater expression of alternative metabolic pathways related to the synthesis of VFAs and pyrimidine nucleotides, possibly linked to bacterial growth. Finally, eight relevant biomarkers were detected and correlated with the synthesis of VFAs.

## Figures and Tables

**Table 1:** Percentage of dry matter (DM) and chemical composition of the feed used for the experiment. The inclusion levels of grass and concentrate were calculated for each lamb to achieve a 100 g/day weight gain.

%	DM [%]	CP [%]	EE [%]	aNDF [%]	ADF [%]	Ash [%]
Grass	29.35	6.97	63.46	37.74	37.74	5.35
Concentrate feed	91.2	17.87	11.49	4.36	4.36	4.25

DM: Dry matter; CP: crude protein; EE: ether extract; aNDF: neutral detergent fiber; and ADF: acid detergent fiber.

**Table 2:** Average and SEM intake in g/kg dry matter (DM) of both groups at each period of the study.

	Group	DM	*p*	CP	*p*	ADF	*p*	aNDF	*p*
Stabilization	CG	620.50 ± 38.86 ^A^	0.3123	68.12 ± 5.18 ^A^	0.5758	165.69 ± 12.37 ^A^	0.6650	292.26 ± 21.82 ^A^	0.6650
	EG	616.47 ± 34.93 ^A^		67.75 ± 2.12 ^A^		164.32 ± 6.42 ^A^		289.85 ± 11.32 ^A^	
DPS14	CG	639.52 ± 21.81 ^A^	0.11233	69.25 ± 2.28 ^A^	0.4974	168.12 ± 4.63 ^A^	0.0303	296.54 ± 8.17 ^A^	0.0303
	EG	736.20 ± 31.96 ^A^		70.37 ± 3.29 ^A^		246.78 ± 1.61 ^B^		414.51 ± 2.72 ^B^	
DPS44	CG	692.02 ± 22.66 ^A^	0.1123	135.66 ± 3.08 ^A^	0.0303	150.81 ± 7.62 ^A^	0.0303	312.98 ± 9.49 ^A^	0.0303
	EG	746.20 ± 21.93 ^A^		135.16 ± 1.25 ^B^		245.55 ± 0.89 ^B^		412.44 ± 1.50 ^B^	

DM: dry matter; CP: crude protein; aNDF: neutral detergent fiber; and ADF: acid detergent fiber. Different letters indicate significant differences by non-parametric Mann–Witney W test for medians (*p* < 0.05).

**Table 3:** Volatile fatty acid concentration in the rumen fluid of lambs during stabilization, and DPS14 and DPS44 periods. Means ± standard errors, significant differences between group treatment and periods (*p* < 0.05) and orthogonal contrasts (*p* < 0.05) are presented.

Productive Parameters	Group	Stabilization	DPS14	DPS44	EE	P Group	P Time	P Interaction	Orthogonal Contrast	
									Linear	Quadratic
Total VFA (mmol/100 mL)	CG	55.56 ± 12.32 ^A–A^	56.57 ± 12.18 ^A–A^	45.61 ± 4.82 ^A–A^	3.3705	0.6472	0.3166	0.7782	0.4119	0.5653
	EG	52.68 ± 2.83 ^A–A^	65.42 ± 9.75 ^A–A^	50.05 ± 1.71 ^A–A^					0.8256	1906
Rumen VFA (molar %)										
			
Acetate	CG	63.98 ± 2.58 ^A–A^	62.50 ± 1.67 ^A–A^	61.30 ± 1.98 ^A–A^	0.8212	0.1146	0.6442	0.5461	0.3644	0.9575
	EG	65.84 ± 2.31 ^A–A^	63.47 ± 1.82 ^A–A^	66.56 ± 0.68 ^A–A^					0.8030	0.2894
Propionate	CG	18.71 ± 2.27 ^A–A^	16.93 ± 2.08 ^A–A^	19.99 ± 1.50 ^A–A^	0.7722	0.6696	0.3924	0.1075	0.6400	0.3167
	EG	14.74 ± 1.86 ^A–A^	21.35 ± 1.71^A–A^	17.93 ± 0.60 ^A–A^					0.2561	0.0513
Butyrate	CG	15.24 ± 1.41 ^A–A^	17.72 ± 1.27 ^A–A^	16.33 ± 1.38 ^A–A^	0.3623	0.14057	0.3294	0.0130	0.4045	0.1008
	EG	16.50 ± 0.89 ^A–A^	13.05 ± 1.57 ^AB–B^	12.68 ± 0.27 ^B–A^					0.0102	0.1800
Iso-butyrate	CG	0.11 ± 0.11 ^A–A^	0.05 ± 0.05 ^A–A^	0.34 ± 0.11 ^A–A^	0.0640	0.0013	0.1645	0.8713	0.3269	0.3955
	EG	0.42 ± 0.18 ^A–A^	0.48 ± 0.02 ^A–A^	0.76 ± 0.22 ^A–A^					0.1423	0.5668
Valerate	CG	2.69 ± 0.56 ^A–B^	1.83 ± 0.33 ^A–A^	1.37 ± 0.44 ^B–A^	0.1275	0.0557	0.1854	0.0907	0.3239	0.0149
	EG	1.30 ± 0.38 ^A–B^	0.95 ± 0.10 ^A–B^	1.04 ± 0.03 ^A–A^					0.5707	0.5807
Iso-valerate	CG	0.12 ± 0.12 ^A–A^	0.16 ± 0.05 ^A–A^	0.49 ± 0.17 ^A–B^	0.0497	0.0004	0.0580	0.0729	0.0577	0.3518
	EG	1.20 ± 0.18 ^AB–B^	0.70 ± 0.07 ^AB–B^	1.01 ± 0.03 ^B–B^					0.3021	0.0189
Acetate: propionate	CG	3.62 ± 0.58 ^A–A^	3.95 ± 0.71 ^A–A^	3.13 ± 0.31 ^A–A^	0.2295	0.5453	0.3433	0.2016	0.5525	0.4203
	EG	4.79 ± 0.88 ^A–A^	3.03 ± 0.27 ^A–A^	3.73 ± 0.15 ^A–A^					0.2060	0.1010

CG: housed; EG: grazing; DPS14: 14 days post-stabilization; and DPS44: 44 days post-stabilization. The first letter of each combination represents the differences between time points within each group. The second letter, separated by a dash, indicates differences between groups within each time point. Same letters indicate no significant differences according to the Bonferroni test (*p* < 0.05).

**Table 4:** Beta diversity analysis of the ruminal microbiome between housed lambs (CG) and grazing lambs (EG) and between periods in both experimental groups (stabilization and DPS44).

		Jaccard		Sokal-Sneath		Yule Y		UniFrac-W		Bray-Curtis	
		Ps-F	*p*	Ps-F	*p*	Ps-F	*p*	Ps-F	*p*	Ps-F	*p*
PERMANOVA		1.33	0.004	1.17	0.003	4.24	0.004	2.63	0.001	1.74	0.001
Pairwise comparison											
CG-s	CG-DPS44	1.05	0.32	1.01	0.35	2.55	0.22	2.45	0.12	1.25	0.36
CG-s	EG-s	1.01	0.42	1.01	0.43	0.93	0.36	0.75	0.51	0.97	0.47
CG-DPS44	EG-DPS44	1.39	0.02	1.21	0.02	4.27	0.02	3.89	0.09	2.02	0.03
EG-s	EG-DPS44	1.64	0.03	1.34	0.03	6.53	0.02	4.10	0.02	2.61	0.03

CG: Control group; EG: Experimental group; s: stabilization; and DPS44: 44 days post-stabilization; Ps-F: Pseudo-F.

**Table 5:** Differential abundance taxonomic levels between EG-s and EG-DPS44 groups. W values indicate the number of comparisons in which each taxon was identified as differentially abundant. Statistical significance of FDR-adjusted *p*-value (*q*) ≤ 0.05.

Taxonomy		EG-s				EG-DPS44				W	*p*	*q*
		25	50	75	100	25	50	75	100			
Phylum	*Verrucomicrobiota*	9.5	16	29.75	53	569.25	1093.5	1482.5	1571	4	3.08 × 10^−05^	4.3 × 10^−04^
	*Actinobacteriota*	52.5	121.5	216.25	307	13.75	18.5	22.5	24	3	7.10 × 10^−04^	4.9 × 10^−03^
Class	*Kiritimatiellae*	1	3	16	49	562.25	1087	1476	1569	4	3.08 × 10^−05^	3.0 × 10^−04^
	*Alphaproteobacteria*	1	1	1	1	6	9.5	35.25	102	4	4.24 × 10^−04^	2.1 × 10^−04^
	*Actinobacteria*	30	105	184.5	198	1	3	6	9	4	8.69 × 10^−06^	1.7 × 10^−04^
	*Negativicutes*	483	589.5	671	731	184.25	211.5	233.5	247	4	1.02 × 10^−04^	6.7 × 10^−04^
	*Bacilli*	232.5	1113	2097.75	2457	33	52.5	81.5	116	3	8.99 × 10^−04^	3.5 × 10^−043^
Order	*WCHB1-41*	1	3	16	49	562.25	1087	1476	1569	4	3.10 × 10^−05^	6.35 × 10^−04^
	*Christensenellales*	230.5	284	419.5	739	744.75	856.5	1155.2	1753	3	6.52 × 10^−03^	4.4 × 10^−02^
	*Rhodospirillales*	1	1	1	1	6	8	33	102	3	6.92 × 10^−04^	7.0 × 10^−03^
	*Erysipelotrichales*	220.75	1091	2069.5	2443	22.75	34	54.75	90	4	2.85 × 10^−04^	3.8 × 10^−03^
	*Bifidobacteriales*	30	105	184	196	1	3	6	9	4	8.70 × 10^−06^	3.57 × 10^−04^
	*Veillonellales-Selenomonadales*	258.75	353	475.5	588	135.25	149.5	164	194	3	3.28 × 10^−03^	2.6 × 10^−02^
Family	*Bacteroidales RF16_group*	39.5	45	46.25	50	592	768	987	1191	10	8.79 × 10^−22^	5.71 × 10^−20^
	*WCHB1-41*	1	3	16	49	562.25	1087	1476	1569	4	3.10 × 10^−05^	6.71 × 10^−04^
	*Erysipelatoclostridiaceae*	219.5	1089.5	2061.7	2424	12.75	20.5	38.25	78	4	3.67 × 10^−04^	5.96 × 10^−03^
	*Bifidobacteriaceae*	30	105	184	196	1	3	6	9	4	8.70 × 10^−06^	2.83 × 10^−04^
	*Muribaculaceae*	236	565	875.75	899	66.25	73.5	93.75	144	3	1.25 × 10^−03^	1.35 × 10^−02^
Genus	*Bacteroidales RF16_group*	39.5	45	46.25	50	592	768	987	1191	10	8.79 × 10^−22^	1.21 × 10^−19^
	*Lachnospiraceae ND3007_group*	1	4	8.25	12	75	90	103	112	5	2.61 × 10^−07^	1.20 × 10^−05^
	*WCHB1-41*	1	3	16	49	562.25	1087	1476	1569	4	3.10 × 10^−05^	6.64 × 10^−04^
	*Roseburia*	1	1	2	5	7.75	8.5	10.25	14	4	3.37 × 10^−05^	6.64 × 10^−04^
	*Rikenellaceae RC9_gut_group*	76.5	108	144	183	270.75	354.5	414.75	426	3	2.4 × 10^−03^	2.59 × 10^−02^
	*Sharpea*	198.25	217.5	724	2239	1	3	5.75	8	6	8.65 × 10^−10^	5.97 × 10^−08^
	*Prevotella_7*	28	33.5	263.25	948	1	1.5	3.25	7	4	4.41 × 10^−04^	7.61 × 10^−03^
	*Bifidobacterium*	30	105	184	196	1	3	6	9	4	8.7 × 10^−06^	3.00 × 10^−04^
	*FD2005*	12	18	37.75	79	1	1	2.25	6	4	3.02 × 10^−05^	6.64 × 10^−04^
	*Erysipelotrichaceae UCG-002*	17	97	553.25	1691	1	1	1.5	3	3	7.97 × 10^−04^	1.09 × 10^−02^
	*Muribaculaceae*	236	565	875.75	899	66.25	73.5	93.75	144	3	1.2 × 10^−03^	1.43 × 10^−02^
	*Anaerovibrio*	22.75	36.5	60	96	1	4	8.5	13	3	1.2 × 10^−03^	1.43 × 10^−02^
	*[Eubacterium]ruminantium_group*	8.5	11	12.75	18	1	1	1	1	3	4.7 × 10^−03^	4.71 × 10^−02^

## Data Availability

https://www.ncbi.nlm.nih.gov/sra/?term=PRJNA1241704 (accessed on 25 March 2025).

## Supplementary Materials

The following supporting information can be downloaded at: https://www.mdpi.com/article/10.3390/ani15111565/s1, Figure S1: Alpha rarefaction curve plotted using Shannon index, which shows how alpha diversity changes as the number of reads in a sample increase; Figure S2: Principal Coordinate Analysis (PCoA) plots based on metrics: Jaccard, Bray–Curtis, weighted and unweighted UniFrac. Analysis was performed between GC and EG groups before and after grazing (stabilization and grazing44); Figure S3: Representative heatmaps of the most abundant bacteria at the genus level: (A) Heatmap with all elements of the CG and EG groups GC and EG groups before and after grazing; (B) Heatmap with all elements of the EGs and EG-DPS44 groups. The dendrogram on the left shows the similarity in abundance between phyla, and the one at the top shows the similarity between the elements of each group. Table S1: Voluntary Intake, and Live Weight Gain (lambs during stabilization, and 14DPS and 14DPS periods. Means ± standard errors, significant differences between periods (*p* < 0.05) and orthogonal contrasts (*p* < 0.05) are presented; Table S2: Filtering and Quality Control Statistics of Sequence Data per Sample; Table S3: Unique taxonomic genera in grazing lambs.

## Abbreviations

The following abbreviations are used in this manuscript:

BW	Body Weight
VFAs	Volatile Fatty Acids
LDF	Low Deciduous Forest
EG	Experimental Group
CG	Control Group
EGs	Experimental Group Stabilization
CGs	Control Group Stabilization
EGg14	Experimental Group grazing 14 days
CGg14	Control Group grazing 14 days
EG-DPS44	Experimental Group grazing 44 days
CGg44	Control Group grazing 44 days
VI	Voluntary Intake
DWG	Daily Weight Gain
TCA	Tricarboxylic Acid Cycle

## References

[1] Sanjorjo R.A., Tseten T., Kang M.-K., Kwon M., Kim S.-W. (2023). In Pursuit of Understanding the Rumen Microbiome. Fermentation.

[2] McDonald P., Edwards R.A., Greenhalgh J.F.D., Morgan C.A., Sinclair L.-A., Wilkinson R.G. (2010). Animal Nutrition.

[3] Keum G.B., Pandey S., Kim E.S., Doo H., Kwak J., Ryu S., Choi Y., Kang J., Kim S., Kim H.B. (2024). Understanding the Diversity and Roles of the Ruminal Microbiome. J. Microbiol..

[4] Henderson G., Cox F., Ganesh S., Jonker A., Young W., Janssen P.H. (2015). Rumen Microbial Community Composition Varies with Diet and Host, but a Core Microbiome Is Found across a Wide Geographical Range. Sci. Rep..

[5] Ye X., Li K., Li Y., Gu M., Omoor I.N.A., Liu H., Qiu S., Jiang X., Lu J., Ma Z. (2024). Deciphering the Impact of Nutrient Composition and Tissue Structure on Rumen Microbiome Dynamics in Roughage Degradation. Res. Artic..

[6] Zhou G., Li J., Liang X., Yang B., He X., Tang H., Guo H., Liu G., Cui W., Chen Y. (2024). Multi-Omics Revealed the Mechanism of Feed Efficiency in Sheep by the Combined Action of the Host and Rumen Microbiota. Anim. Nutr..

[7] Perez H.G., Stevenson C.K., Lourenco J.M., Callaway T.R. (2024). Understanding Rumen Microbiology: An Overview. Encyclopedia.

[8] He S., Yuan Z., Dai S., Wang Z., Zhao S., Wang R., Li Q., Mao H., Wu D. (2024). Intensive Feeding Alters the Rumen Microbiota and Its Fermentation Parameters in Natural Grazing Yaks. Front. Vet. Sci..

[9] Torres-Fajardo R.A., González-Pech P.G., Torres-Acosta J.F.D.J., Sandoval-Castro C.A. (2021). Nutraceutical Potential of the Low Deciduous Forest to Improve Small Ruminant Nutrition and Health: A Systematic Review. Agronomy.

[10] Torres-Fajardo R.A., Ortíz-Domínguez G., González-Pech P.G., Sandoval-Castro C.A., Torres-Acosta J.F.d.J. (2024). The Complexity of Goats’ Feeding Behaviour: An Overview of the Research in the Tropical Low Deciduous Forest. Small Rumin. Res..

[11] Ventura-Cordero J., González-Pech P.G., Torres-Acosta J.F.J., Sandoval-Castro C.A., Tun-Garrido J. (2017). Sheep and Goat Browsing a Tropical Deciduous Forest during the Rainy Season: Why Does Similar Plant Species Consumption Result in Different Nutrient Intake?. Anim. Prod. Sci..

[12] Belanche A., Kingston-Smith A.H., Griffith G.W., Newbold C.J. (2019). A Multi-Kingdom Study Reveals the Plasticity of the Rumen Microbiota in Response to a Shift From Non-Grazing to Grazing Diets in Sheep. Front. Microbiol..

[13] Guo J., Li P., Liu S., Miao B., Zeng B., Jiang Y., Li L., Wang L., Chen Y., Zhang H. (2020). Characterization of the Rumen Microbiota and Volatile Fatty Acid Profiles of Weaned Goat Kids under Shrub-Grassland Grazing and Indoor Feeding. Animals.

[14] Lv F., Wang X., Pang X., Liu G. (2020). Effects of Supplementary Feeding on the Rumen Morphology and Bacterial Diversity in Lambs. PeerJ.

[15] Van Gylswyk N.O. (1970). The Effect of Supplementing a Low–Protein Hay on the Cellulolytic Bacteria in the Rumen of Sheep and on the Digestibility of Cellulose and Hemicellulose. J. Agric. Sci..

[16] Hespell R.B., Wolf R., Bothast R.J. (1987). Fermentation of Xylans by Butyrivibrio Fibrisolvens and Other Ruminal Bacteria. Appl. Environ. Microbiol..

[17] Barraza A., Montes-Sánchez J.J., Caamal-Chan M.G., Loera-Muro A. (2021). Characterization of Microbial Communities from Rumen and Large Intestine of Lactating Creole Goats Grazing in Arid Plant Communities. Microbiology.

[18] González-Pech P.G., Marín-Tun C.G., Valladares-González D.A., Ventura-Cordero J., Ortiz-Ocampo G.I., Cámara-Sarmiento R., Sandoval-Castro C.A., Torres-Acosta J.F.J. (2018). A Protocol of Human Animal Interaction to Habituate Young Sheep and Goats for Behavioural Studies. Behav. Process..

[19] Agreil C., Meuret M. (2004). An Improved Method for Quantifying Intake Rate and Ingestive Behaviour of Ruminants in Diverse and Variable Habitats Using Direct Observation. Small Rumin. Res..

[20] Gonzalez-Pech P.G., Torres-Acosta J.F.J., Castro C.A.S. (2014). Adapting a bite coding grid for small ruminants browsing a deciduous tropical forest. Trop. Subtrop. Agroecosyst..

[21] Martín E., Pérez E., Cañón S., Rodríguez J., Rodriguez F.d.S. (2005). Sonda oro-ruminal experimental como alternativa para la obtención de microorganismos anaeróbicos del rumen. Cienc. Tecnol. Agropecu..

[22] Barros-Rodríguez M.A., Solorio-Sánchez F.J., Sandoval-Castro C.A., Klieve A., Rojas-Herrera R.A., Briceño-Poot E.G., Ku-Vera J.C. (2015). Rumen Function In Vivo and In Vitro in Sheep Fed *Leucaena leucocephala*. Trop. Anim. Health Prod..

[23] Rodríguez G.F., Llamas L.G., Castellanos R.A., Llamas L.G., Shimada A.S., Sistema de educación continua en producción animal en México (1990). Manual de Técnicas de Investigación En Rumiología. Digestibilidad, Balance de Nutrimentos y Patrones de Fermentación Ruminal.

[24] Ryan J.P. (1980). Determination of Volatile Fatty Acids and Some Related Compounds in Ovine Rumen Fluid, Urine, and Blood Plasma, by Gas-Liquid Chromatography. Anal. Biochem..

[25] Winer B.J. (1971). Statistical Principles in Experimental Design.

[26] Kuehl R.O. (2001). Diseño de Experimentos: Principios Estadísticos de Diseño Y Análisis De Investigación.

[27] Milliken G.A., Johnson D.E. (2009). Analysis of Messy Data.

[28] Callahan B.J., McMurdie P.J., Rosen M.J., Han A.W., Johnson A.J.A., Holmes S.P. (2016). DADA2: High-Resolution Sample Inference from Illumina Amplicon Data. Nat. Methods.

[29] Bokulich N.A., Kaehler B.D., Rideout J.R., Dillon M., Bolyen E., Knight R., Huttley G.A., Gregory Caporaso J. (2018). Optimizing Taxonomic Classification of Marker-Gene Amplicon Sequences with QIIME 2′s Q2-Feature-Classifier Plugin. Microbiome.

[30] Kaehler B.D., Bokulich N.A., McDonald D., Knight R., Caporaso J.G., Huttley G.A. (2019). Species Abundance Information Improves Sequence Taxonomy Classification Accuracy. Nat. Commun..

[31] Jaccard P. (1908). Nouvelles Recherches Sur La Distribution Florale. Bull. Soc. Vaud. Sci. Nat..

[32] Sokal R.R. (1963). The Principles and Practice of Numerical Taxonomy. Taxon.

[33] Fisher R.A., Corbet A.S., Williams C.B. (1943). The Relation Between the Number of Species and the Number of Individuals in a Random Sample of an Animal Population. J. Anim. Ecol..

[34] Lozupone C.A., Hamady M., Kelley S.T., Knight R. (2007). Quantitative and Qualitative Beta Diversity Measures Lead to Different Insights into Factors That Structure Microbial Communities. Appl. Environ. Microbiol..

[35] Sørensen T. (1948). A Method of Establishing Groups of Equal Amplitude in Plant Sociology Based on Similarity of Species Content and Its Application to Analyses of the Vegetation on Danish Commons.

[36] Bolyen E., Rideout J.R., Dillon M.R., Bokulich N.A., Abnet C.C., Al-Ghalith G.A., Alexander H., Alm E.J., Arumugam M., Asnicar F. (2019). Reproducible, Interactive, Scalable and Extensible Microbiome Data Science Using QIIME 2. Nat. Biotechnol..

[37] McMurdie P.J., Holmes S. (2013). Phyloseq: An R Package for Reproducible Interactive Analysis and Graphics of Microbiome Census Data. PLoS ONE.

[38] Lin H., Peddada S.D. (2020). Analysis of Compositions of Microbiomes with Bias Correction. Nat. Commun..

[39] Douglas G.M., Maffei V.J., Zaneveld J.R., Yurgel S.N., Brown J.R., Taylor C.M., Huttenhower C., Langille M.G.I. (2020). PICRUSt2 for Prediction of Metagenome Functions. Nat. Biotechnol..

[40] Yang C., Mai J., Cao X., Burberry A., Cominelli F., Zhang L. (2023). Ggpicrust2: An R Package for PICRUSt2 Predicted Functional Profile Analysis and Visualization. Bioinformatics.

[41] Caspi R., Billington R., Keseler I.M., Kothari A., Krummenacker M., Midford P.E., Ong W.K., Paley S., Subhraveti P., Karp P.D. (2020). The MetaCyc Database of Metabolic Pathways and Enzymes—A 2019 Update. Nucleic Acids Res..

[42] Mallick H., Rahnavard A., McIver L.J., Ma S., Zhang Y., Nguyen L.H., Tickle T.L., Weingart G., Ren B., Schwager E.H. (2021). Multivariable Association Discovery in Population-Scale Meta-Omics Studies. PLoS Comput. Biol..

[43] Duncan A.J., Poppi D.P., Gordon I.J., Prins H.H.T. (2008). Nutritional Ecology of Grazing and Browsing Ruminants. The Ecology of Browsing and Grazing.

[44] Sanon H.O., Kaboré-Zoungrana C., Ledin I. (2007). Behaviour of Goats, Sheep and Cattle and Their Selection of Browse Species on Natural Pasture in a Sahelian Area. Small Rumin. Res..

[45] Janis C., Gordon I.J., Prins H.H.T. (2008). An Evolutionary History of Browsing and Grazing Ungulates. The Ecology of Browsing and Grazing.

[46] Jaimez-Rodríguez P.R., González-Pech P.G., Ventura-Cordero J., Brito D.R.B., Costa-Júnior L.M., Sandoval-Castro C.A., Torres-Acosta J.F.J. (2019). The Worm Burden of Tracer Kids and Lambs Browsing Heterogeneous Vegetation Is Influenced by Strata Harvested and Not Total Dry Matter Intake or Plant Life Form. Trop. Anim. Health Prod..

[47] González-Pech P.G., Torres-Acosta J.F.D.J., Sandoval-Castro C.A., Tun-Garrido J. (2015). Feeding Behavior of Sheep and Goats in a Deciduous Tropical Forest during the Dry Season: The Same Menu Consumed Differently. Small Rumin. Res..

[48] González-Pech P.G., Ventura-Cordero J., Ortiz-Ocampo G.I., Jaimez-Rodríguez P.R., Tun Garrido J.D.L.C., Sandoval-Castro C.A., Torres-Acosta J.F.D.J. (2017). Plantas Consumidas Por Ovinos y Caprinos En La Selva Baja Caducifolia de Yucatán. Guía Ilustrada.

[49] Camacho-Escobar M.A., Galicia-Jiménez M.M., Sánchez-Bernal E.I., Ávila-Serrano N.Y., López-Garrido S.J., Camacho-Escobar M.A., Galicia-Jiménez M.M., Sánchez-Bernal E.I., Ávila-Serrano N.Y., López-Garrido S.J. (2020). Producción de metano y bióxido de carbono *in vitro* de pastos tropicales de la costa de Oaxaca, México. Terra Latinoam..

[50] López Herrera M., Arias Gamboa M., Alpizar Naranjo A., Castillo Umaña M. (2021). Calidad de fibra y producción de metano en ensilados de leguminosas con fuentes de carbohidratos. Nutr. Anim. Trop..

[51] Nozière P., Ortigues-Marty I., Loncke C., Sauvant D. (2010). Carbohydrate Quantitative Digestion and Absorption in Ruminants: From Feed Starch and Fibre to Nutrients Available for Tissues. Animal.

[52] Zhang C., Lian Z., Xu B., Shen Q., Bao M., Huang Z., Jiang H., Li W. (2023). Gut Microbiome Variation Along A Lifestyle Gradient Reveals Threats Faced by Asian Elephants. Genom. Proteom. Bioinform..

[53] Guo N., Wu Q., Shi F., Niu J., Zhang T., Degen A.A., Fang Q., Ding L., Shang Z., Zhang Z. (2021). Seasonal Dynamics of Diet–Gut Microbiota Interaction in Adaptation of Yaks to Life at High Altitude. Npj Biofilms Microbiomes.

[54] Desai M.S., Seekatz A.M., Koropatkin N.M., Kamada N., Hickey C.A., Wolter M., Pudlo N.A., Kitamoto S., Terrapon N., Muller A. (2016). A Dietary Fiber-Deprived Gut Microbiota Degrades the Colonic Mucus Barrier and Enhances Pathogen Susceptibility. Cell.

[55] Derrien M., Vaughan E.E., Plugge C.M., de Vos W.M. (2004). Akkermansia Muciniphila Gen. Nov., Sp. Nov., a Human Intestinal Mucin-Degrading Bacterium. Int. J. Syst. Evol. Microbiol..

[56] Wei H., Ding L., Wang X., Yan Q., Jiang C., Hu C., Wang G., Zhou Y., Henkin Z., Degen A.A. (2021). Astragalus Root Extract Improved Average Daily Gain, Immunity, Antioxidant Status and Ruminal Microbiota of Early Weaned Yak Calves. J. Sci. Food Agric..

[57] Ghedini C.P., Silva L.H.P., Moura D.C., Brito A.F. (2024). Supplementing Flaxseed Meal with Sucrose, Flaxseed Oil, or Both: Effects on Milk Enterolactone, Ruminal Microbiota Profile, Production Performance, and Nutrient Utilization in Dairy Cows. J. Dairy Sci..

[58] Qiu Q., Zhang J., Qu M., Li Y., Zhao X., Ouyang K. (2023). Effect of Energy Provision Strategy on Rumen Fermentation Characteristics, Bacterial Diversity and Community Composition. Bioengineering.

[59] Wang Y., Nan X., Zhao Y., Jiang L., Wang H., Zhang F., Hua D., Liu J., Yao J., Yang L. (2021). Dietary Supplementation of Inulin Ameliorates Subclinical Mastitis via Regulation of Rumen Microbial Community and Metabolites in Dairy Cows. Microbiol. Spectr..

[60] El Otmani S., Chebli Y., Taminiau B., Chentouf M., Hornick J.-L., Cabaraux J.-F. (2021). Effect of Olive Cake and Cactus Cladodes Incorporation in Goat Kids’ Diet on the Rumen Microbial Community Profile and Meat Fatty Acid Composition. Biology.

[61] Ma S., Zhang Y., Li Z., Guo M., Liu B., Wang Z., Cui Y., Wang C., Li D., Shi Y. (2024). Roughage Quality Determines the Production Performance of Post-Weaned Hu Sheep via Altering Ruminal Fermentation, Morphology, Microbiota, and the Global Methylome Landscape of the Rumen Wall. Front. Microbiomes.

[62] Zhang X., Zhao Y., Zhang M., Pang X., Xu J., Kang C., Li M., Zhang C., Zhang Z., Zhang Y. (2012). Structural Changes of Gut Microbiota during Berberine-Mediated Prevention of Obesity and Insulin Resistance in High-Fat Diet-Fed Rats. PLoS ONE.

[63] McLoughlin S., Spillane C., Campion F.P., Claffey N., Sosa C.C., McNicholas Y., Smith P.E., Diskin M.G., Waters S.M. (2023). Breed and Ruminal Fraction Effects on Bacterial and Archaeal Community Composition in Sheep. Sci. Rep..

[64] Yin X., Ji S., Duan C., Tian P., Ju S., Yan H., Zhang Y., Liu Y. (2021). Age-Related Changes in the Ruminal Microbiota and Their Relationship With Rumen Fermentation in Lambs. Front. Microbiol..

[65] Schwab C.G., Broderick G.A. (2017). A 100-Year Review: Protein and Amino Acid Nutrition in Dairy Cows. J. Dairy Sci..

[66] Wang D., Tang G., Wang Y., Yu J., Chen L., Chen J., Wu Y., Zhang Y., Cao Y., Yao J. (2023). Rumen Bacterial Cluster Identification and Its Influence on Rumen Metabolites and Growth Performance of Young Goats. Anim. Nutr..

[67] Li L., Qu J., Zhu H., Liu Y., Wu J., Shao G., Guan X., Qu Y. (2024). Effects of Feeding Different Levels of Dietary Corn Silage on Growth Performance, Rumen Fermentation and Bacterial Community of Post-Weaning Dairy Calves. Anim. Biosci..

[68] Schären M., Frahm J., Kersten S., Meyer U., Hummel J., Breves G., Dänicke S. (2018). Interrelations between the Rumen Microbiota and Production, Behavioral, Rumen Fermentation, Metabolic, and Immunological Attributes of Dairy Cows. J. Dairy Sci..

[69] Khiaosa-ard R., Mahmood M., Mickdam E., Pacífico C., Meixner J., Traintinger L.-S. (2023). Winery By-Products as a Feed Source with Functional Properties: Dose–Response Effect of Grape Pomace, Grape Seed Meal, and Grape Seed Extract on Rumen Microbial Community and Their Fermentation Activity in RUSITEC. J. Anim. Sci. Biotechnol..

[70] Wang S., Chai J., Zhao G., Zhang N., Cui K., Bi Y., Ma T., Tu Y., Diao Q. (2022). The Temporal Dynamics of Rumen Microbiota in Early Weaned Lambs. Microorganisms.

[71] Pang J., Liu L., Liu X., Wang Y., Chen B., Wu S., Yao J., Xu X. (2021). A Novel Identified Pseudomonas Aeruginosa, Which Exhibited Nitrate- and Nitrite-Dependent Methane Oxidation Abilities, Could Alleviate the Disadvantages Caused by Nitrate Supplementation in Rumen Fluid Fermentation. Microb. Biotechnol..

[72] Stackebrandt E.S., Hippie H. (2015). Ruminobacter. Bergey’s Manual of Systematics of Archaea and Bacteria.

[73] Zhou M., Yan B., Wong J.W.C., Zhang Y. (2018). Enhanced Volatile Fatty Acids Production from Anaerobic Fermentation of Food Waste: A Mini-Review Focusing on Acidogenic Metabolic Pathways. Bioresour. Technol..

[74] Dewhirst F.E., Paster B.J., Fontaine S.L., Rood J.I. (1990). Transfer of Kingella Indologenes (Snell and Lapage 1976) to the Genus *Suttonella* Gen. Nov. as *Suttonella Indologenes* Comb. Nov.; Transfer of Bacteroides Nodosus (Beveridge 1941) to the Genus Dichelobacter Gen. Nov. as Dichelobacter Nodosus Comb. Nov.; and Assignment of the Genera Cardiobacterium, Dichelobacter, and Suttonella to Cardiobacteriaceae Fam. Nov. in the Gamma Division of Proteobacteria on the Basis of 16S rRNA Sequence Comparisons. Int. J. Syst. Evol. Microbiol..

[75] Zhang Y., Zhang X., Li C., Tian H., Weng X., Lin C., Zhang D., Zhao Y., Li X., Cheng J. (2024). Rumen Microbiome and Fat Deposition in Sheep: Insights from a Bidirectional Mendelian Randomization Study. Npj Biofilms Microbiomes.

[76] Ma T., Wu W., Tu Y., Zhang N., Diao Q. (2020). Resveratrol Affects in Vitro Rumen Fermentation, Methane Production and Prokaryotic Community Composition in a Time- and Diet-Specific Manner. Microb. Biotechnol..

[77] Dai H., Huang Q., Li S., Du D., Yu W., Guo J., Zhao Z., Yu X., Ma F., Sun P. (2024). Effect of Dietary Benzoic Acid Supplementation on Growth Performance, Rumen Fermentation, and Rumen Microbiota in Weaned Holstein Dairy Calves. Animals.

[78] Li C., Chen N., Zhang X., Shahzad K., Qi R., Zhang Z., Lu Z., Lu Y., Yu X., Zafar M.H. (2022). Mixed Silage with Chinese Cabbage Waste Enhances Antioxidant Ability by Increasing Ascorbate and Aldarate Metabolism through Rumen Prevotellaceae UCG-004 in Hu Sheep. Front. Microbiol..

[79] Shen J., Li Z., Yu Z., Zhu W. (2020). Effects of Dietary Replacement of Soybean Meal with Dried Distillers Grains with Solubles on the Microbiota Occupying Different Ecological Niches in the Rumen of Growing Hu Lambs. J. Anim. Sci. Biotechnol..

[80] Zhang H., Elolimy A.A., Akbar H., Thanh L.P., Yang Z., Loor J.J. (2022). Association of Residual Feed Intake with Peripartal Ruminal Microbiome and Milk Fatty Acid Composition during Early Lactation in Holstein Dairy Cows. J. Dairy Sci..

[81] Hao Y., Ouyang T., Wang W., Wang Y., Cao Z., Yang H., Guan L.L., Li S. (2024). Competitive Analysis of Rumen and Hindgut Microbiota Composition and Fermentation Function in Diarrheic and Non-Diarrheic Postpartum Dairy Cows. Microorganisms.

[82] Cusa E., Obradors N., Baldomà L., Badía J., Aguilar J. (1999). Genetic Analysis of a Chromosomal Region Containing Genes Required for Assimilation of Allantoin Nitrogen and Linked Glyoxylate Metabolism in *Escherichia coli*. J. Bacteriol..

[83] Rabin R., Reeves H.C., Wegener W.S., Megraw R.E., Ajl S.J. (1965). Glyoxylate in Fatty-Acid Metabolism. Science.

[84] Li Y., Peng R., Kunz C., Wang K., Terranova M., Zhang Y., Macsai M., Frossard E., Niu M. (2024). Hydroponic Fodders as Alternative Feeds for Ruminants to Reduce Ruminal Methane Emissions: An *in Vitro* Study. J. Dairy Sci..

[85] Dessalegn G., Tesfay G. (2015). The Role of Bacteria in Nitrogen Metabolism in the Rumen with Emphasis of Cattle. Res. J. Agric. Environ. Manag..

[86] Russell J.B., O’Connor J.D., Fox D.G., Van Soest P.J., Sniffen C.J. (1992). A Net Carbohydrate and Protein System for Evaluating Cattle Diets: I. Ruminal Fermentation. J. Anim. Sci..

[87] Baldwin J.E., Krebs H. (1981). The Evolution of Metabolic Cycles. Nature.

[88] Kornberg H.L., Krebs H.A. (1957). Synthesis of Cell Constituents from C2-Units by a Modified Tricarboxylic Acid Cycle. Nature.

[89] Liu Z., Jiang A., Lv X., Zhou C., Tan Z. (2024). Metabolic Changes in Serum and Milk of Holstein Cows in Their First to Fourth Parity Revealed by Biochemical Analysis and Untargeted Metabolomics. Anim. Open Access J..

[90] Eastmond P.J., Graham I.A. (2001). Re-Examining the Role of the Glyoxylate Cycle in Oilseeds. Trends Plant Sci..

[91] Asanuma N., Iwamoto M., Hino T. (1999). The Production of Formate, a Substrate for Methanogenesis, from Compounds Related with the Glyoxylate Cycle by Mixed Ruminal Microbes. Nihon Chikusan Gakkaiho.

[92] Zrenner R., Stitt M., Sonnewald U., Boldt R. (2006). Pyrimidine and Purine Biosynthesis and Degradation in Plants. Annu. Rev. Plant Biol..

[93] Kheirandish P., Petri R.M., Sener-Aydemir A., Schwartz-Zimmermann H.E., Berthiller F., Zebeli Q., Pacífico C. (2022). Characterization of Microbial Intolerances and Ruminal Dysbiosis towards Different Dietary Carbohydrate Sources Using an In Vitro Model. J. Appl. Microbiol..

