# Supplementary figures and images for: Effects of Grazing in a Low Deciduous Forest on Rumen Microbiota and Volatile Fatty Acid Production in Lambs
Source: Animals (Basel). 2025 May 27;15(11):1565. doi: 10.3390/ani15111565 (PMC12153761; doi:10.3390/ani15111565)

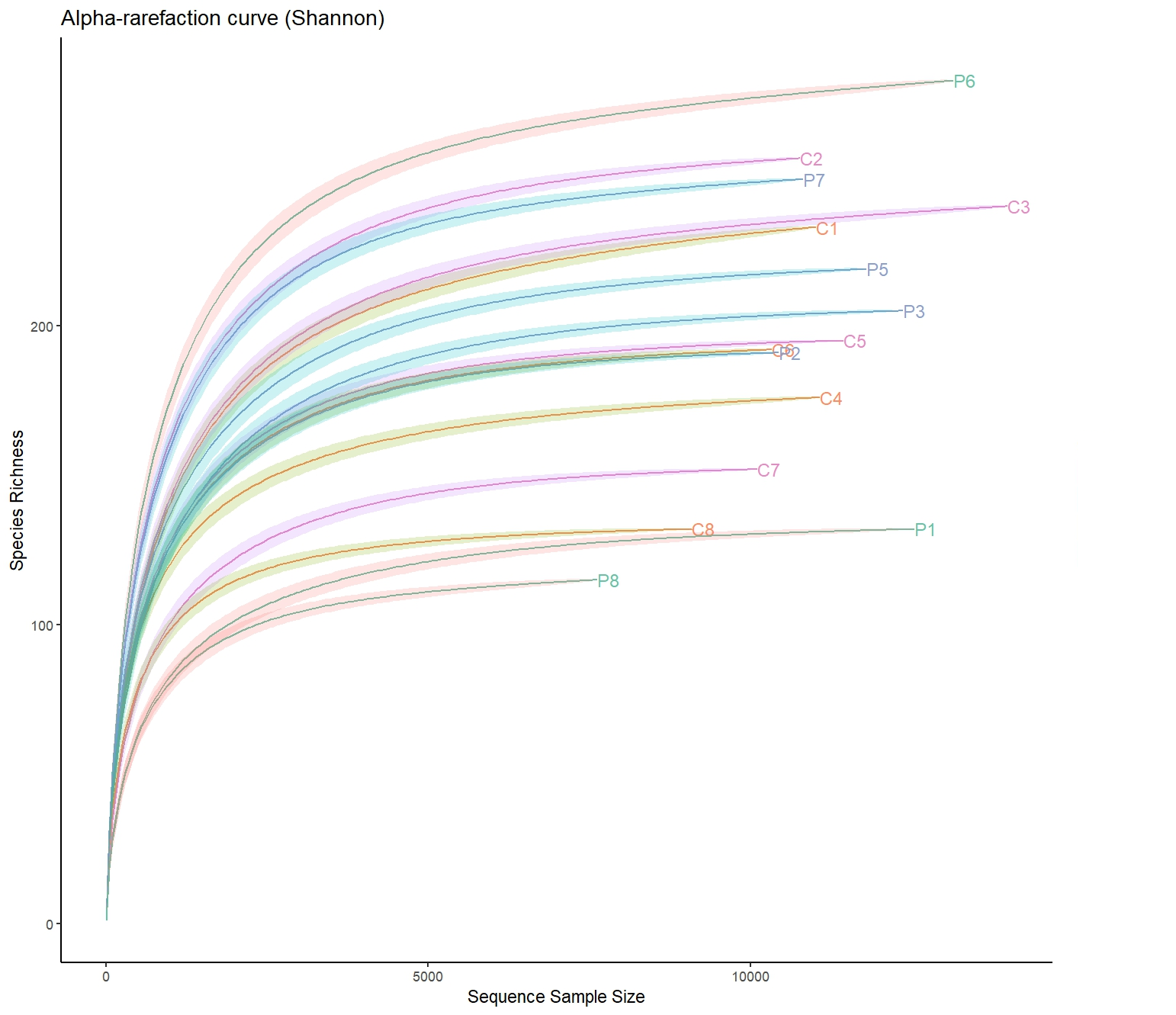

Supplement: Supplementary file 1 [file animals-15-01565-s001.zip › Figure S1. Alpha rarefaction curve plotted using Shannon index.tif]

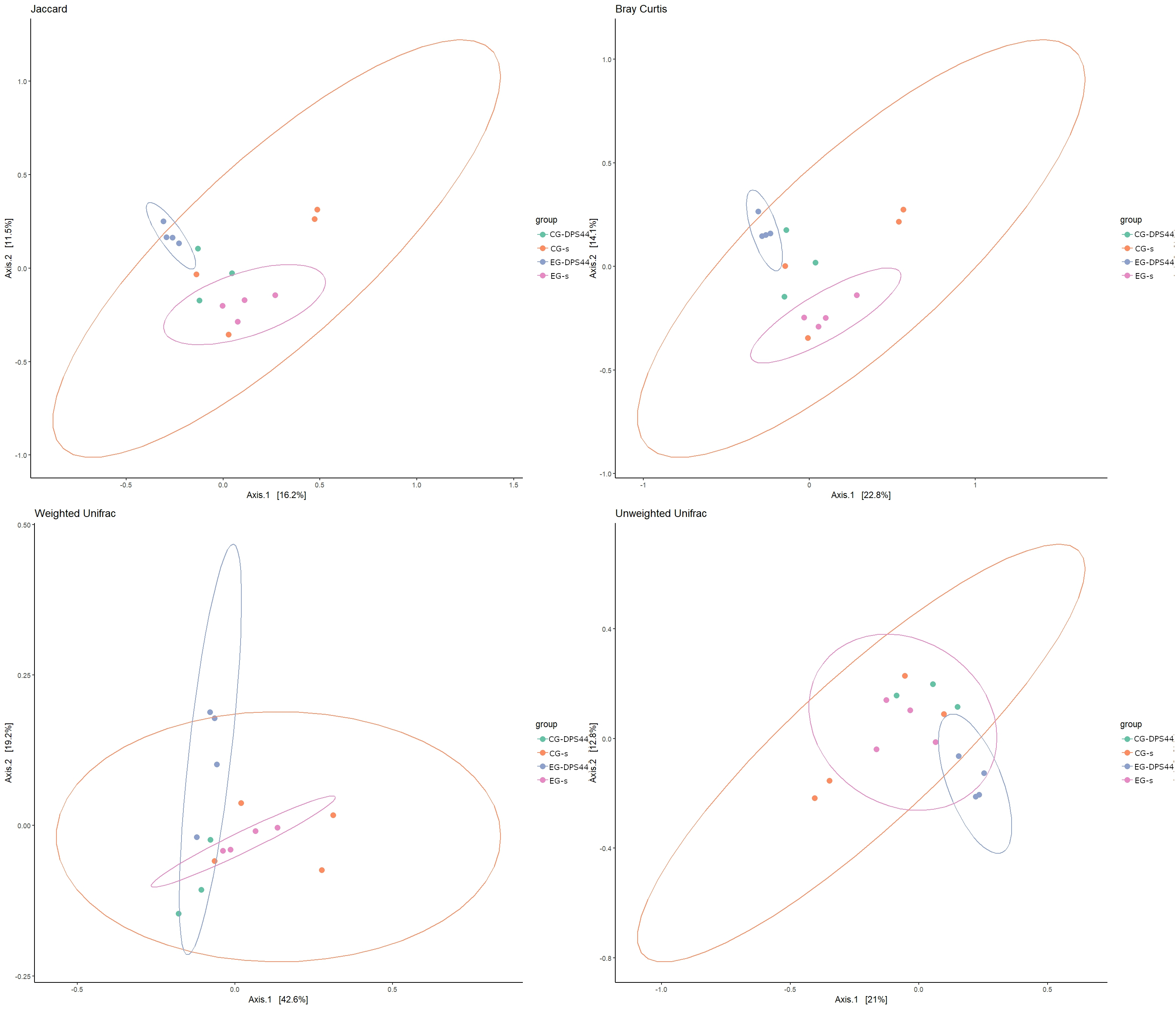

Supplement: Supplementary file 1 [file animals-15-01565-s001.zip › Figure S2. Principal Coordinate Analysis (PCoA) plots based on beta diversity metrics.tif]

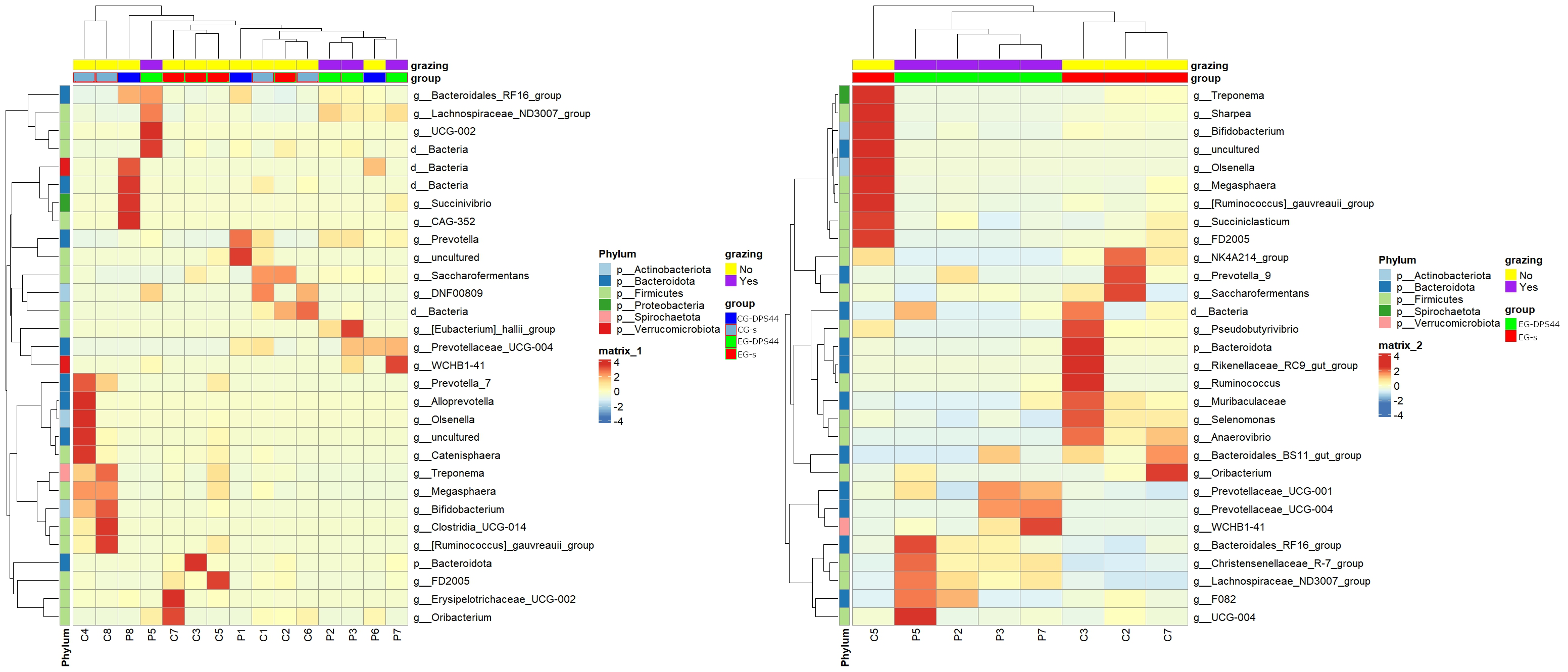

Supplement: Supplementary file 1 [file animals-15-01565-s001.zip › Figure S3. Representative heatmaps of the most abundant bacteria at the genus level A and B.tif]
